# Genomic epidemiology of *Mycobacterium tuberculosis* in Santa Catarina, Southern Brazil

**DOI:** 10.1038/s41598-020-69755-9

**Published:** 2020-07-30

**Authors:** Mirela Verza, Mara Cristina Scheffer, Richard Steiner Salvato, Marcos André Schorner, Fernando Hartmann Barazzetti, Hanalydia de Melo Machado, Taiane Freitas Medeiros, Darcita Buerger Rovaris, Isabel Portugal, Miguel Viveiros, João Perdigão, Afrânio Kritski, Maria Luiza Bazzo

**Affiliations:** 1 Programa Acadêmico de Tuberculose da Faculdade de Medicina e Complexo Hospitalar HUCFF-IDT, Universidade Federal do Rio de Janeiro (UFRJ), Rua Prof. Rodolpho Paulo Rocco, 255, Ilha do Fundão, Rio de Janeiro, RJ 21941-590 Brazil; 2 Laboratório de Biologia Molecular, Microbiologia e Sorologia, Centro de Ciências da Saúde, Universidade Federal de Santa Catarina (UFSC), Florianópolis, Santa Catarina Brazil; 3 Programa de Pós-graduação em Biologia Celular e Molecular, Universidade Federal do Rio Grande do Sul (UFRGS), Porto Alegre, Rio Grande do Sul Brazil; 4 Centro de Desenvolvimento Científico e Tecnológico (CDCT), Centro Estadual de Vigilância em Saúde, Secretaria Estadual da Saúde do Rio Grande do Sul, Av. Ipiranga, 5400, Porto Alegre, RS 90610-000 Brazil; 5 Setor de Bacteriologia da Tuberculose, Laboratório Central do Estado de Santa Catarina (LACEN-SC), Florianópolis, Santa Catarina Brazil; 6 iMed.Ulisboa-Research Institute for Medicines, Faculdade de Farmácia, Universidade de Lisboa, Lisboa, Portugal; 7 Unidade de Microbiologia Médica, Global Health and Tropical Medicine, Instituto de Higiene e Medicina Tropical, Universidade Nova de Lisboa, Lisboa, Portugal

**Keywords:** Molecular biology, Microbiology, Clinical microbiology, Microbial genetics

## Abstract

*Mycobacterium tuberculosis* (*M.tb*), the pathogen responsible for tuberculosis (TB) poses as the major cause of death among infectious diseases. The knowledge about the molecular diversity of *M.tb* enables the implementation of more effective surveillance and control measures and, nowadays, Whole Genome Sequencing (WGS) holds the potential to produce high-resolution epidemiological data in a high-throughput manner. Florianópolis, the state capital of Santa Catarina (SC) in south Brazil, shows a high TB incidence (46.0/100,000). Here we carried out a WGS-based evaluation of the *M.tb* strain diversity, drug-resistance and ongoing transmission in the capital metropolitan region. Resistance to isoniazid, rifampicin, streptomycin was identified respectively in 4.0% (n = 6), 2.0% (n = 3) and 1.3% (n = 2) of the 151 studied strains by WGS. Besides, resistance to pyrazinamide and ethambutol was detected in 0.7% (n = 1) and reistance to ethionamide and fluoroquinolone (FQ) in 1.3% (n = 2), while a single (0.7%) multidrug-resistant (MDR) strain was identified. SNP-based typing classified all isolates into *M.tb* Lineage 4, with high proportion of sublineages LAM (60.3%), T (16.4%) and Haarlem (7.9%). The average core-genome distance between isolates was 420.3 SNPs, with 43.7% of all isolates grouped across 22 genomic clusters thereby showing the presence of important ongoing TB transmission events. Most clusters were geographically distributed across the study setting which highlights the need for an urgent interruption of these large transmission chains. The data conveyed by this study shows the presence of important and uncontrolled TB transmission in the metropolitan area and provides precise data to support TB control measures in this region.

## Introduction

Tuberculosis (TB) remains a major public health threat and is currently the tenth leading cause of death worldwide and the leading cause of death by a single infectious microorganism. In 2018, 1.5 million people died from TB and 10 million new cases are estimated to have occurred worldwide. Brazil, along with the Russian Federation, India, China and South Africa—the BRICS countries—account for more than 40% of the global TB disease burden in incidence and deaths, and about 58% of the global burden of drug-resistant TB. In Brazil, 90,527 TB cases were reported (45 per 100,000 population) in 2018^1^. While the state of Santa Catarina in Southern Brazil is characterized by an intermediate TB incidence rate—23.7 per 100,000 population, the state capital Florianópolis shows a higher incidence rate of 46.0 per 100,000 population along with high rates of TB-HIV co-infection^2^.

Worldwide, the asymmetrical distribution of the disease is also linked with distinctive lineages and genotypes of the *Mycobacterium tuberculosis* (*M.tb*) Complex species, the etiological agent of TB^3^. Early strain typing methods greatly boosted our understanding of TB transmission dynamics while simultaneously providing additional tools and perspectives on the phylogeographic landscape of such strains at a global level^3^. These so-called classical typing methods, i.e., those based on the characterization of genetic repetitive elements, have been successful in informing public health interventions due to the inability of traditional contact tracing approaches to reconstruct complex transmission chains^4–6^. In fact, population-based typing of *M.tb* clinical isolates has been important to infer on the degree of recent infection and highly relevant in the identification of specific risk factors and geospatial hotspots for intensified TB transmission. Two classical examples of such studies are those conducted in San Francisco and New York in the late 1980s and early 1990s that enabled the correlation of specific ethnicities, drug resistance and/or residence in specific areas with recent TB transmission as assessed by genetic clustering based on profiles obtained with Restriction Fragment Length Polymorphisms with IS*6110*^6,7^. In the next decades, classical genotyping methods such as Spoligotyping and Mycobacterial Interspersed Repetitive Unit—Variable Number of Tandem Repeat (MIRU-VNTR) have been widely used for molecular epidemiology studies^8^. While these methods have been an integral part of several national TB control plans, most of the genomic diversity and distinctiveness of each isolate is overlooked by typing methods that are only able to interrogate a minor fraction of an isolate’s genome^9–11^. In contrast, Whole Genome Sequencing (WGS) has emerged as the leading typing strategy to study the dissemination and transmission dynamics at a genome-wide SNP-based resolution that clearly outperforms classical typing methods and, coupled with molecular evolutionary approaches, also holds the potential to retrace the evolutionary history of locally circulating strains^12,13^. This level of resolution has enabled the reconstruction of the microevolutionary trajectory of circulating extensively drug resistant strains in Portugal and South Africa along with the breakdown into genomic clusters that are proposed to reflect epidemiological links between patients^14–16^.

In Santa Catarina state, in Brazil, it is known that the *M.tb* population structure is mostly dominated by Latin American and Mediterranean (LAM) strains, followed by the “ill-defined” T strains as assessed by spoligotyping^17^. However, nothing is known regarding the genomic diversity associated with this region and reports using higher resolution typing methods are limited to 12-loci MIRU-VNTR^18^. To address this gap, herein we report the preliminary results from a genomic epidemiological study in the metropolitan area, aiming to unravel the *M.tb* genotypes circulating in this region, infer on existing and active transmission clusters in the community, and examine the distribution of drug resistance while exploring possible associations between unfavorable treatment outcomes and SNP-based lineages.

## Results

### Study sample and epidemiology

The study encompasses 151 patients diagnosed with TB that started on anti-TB treatment between May 2014 and May 2016 in the metropolitan area. Ninety-six (63.6%) patients were male and 49% of all patients were aged between 26 and 45 years. Regarding the risk factors and comorbidities, 13 (8.6%) patients had diabetes mellitus, 47 (31.1%) had alcohol abuse history, 85 (56.3%) were tobacco users and 47 (31.1%) illicit drug users. Twenty-seven (17.9%) patients were co-infected with human immunodeficiency virus (HIV) (Supplementary Table S1). In addition, nine (6.0%) individuals were homeless and four (2.6%) spent time in a prison establishment.

### Drug resistance and molecular basis

**Figure 1:**
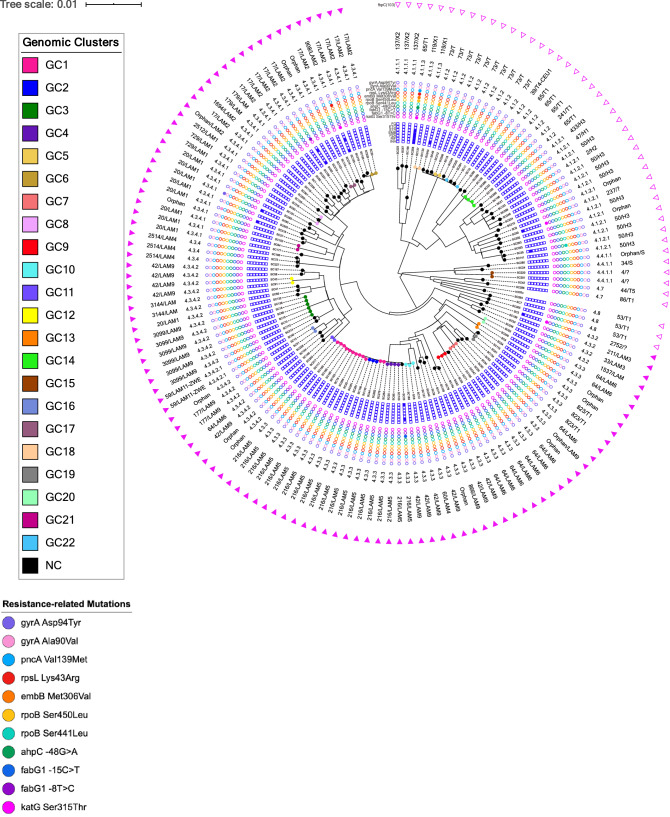
Maximum likelihood phylogenetic tree for the 151 *M.tb* isolates included in the study. This tree was constructed based on 17,027 core SNPs and is shown annotated with (from the outside to the inside): presence or absence of *fbpC*^103^ LAM marker; SIT and spoligotyping clade; SNP-based sub-lineage; drug resistance associated mutations; and genotypic-based prediction for drug susceptibility. Tips are shown coloured according to the genomic cluster (see legend). *NC* not clustered, *GC* genomic cluster.

Among the 151 isolates obtained for the study, genotypic-based prediction for drug resistance enabled the identification of six (4.0%) isoniazid resistant isolates, three (2.0%) with rifampicin resistance, two (1.3%) streptomycin resistant isolates and, resistance to pyrazinamide and ethambutol was detected in one isolate (0.7%). Besides, resistance to second-line drugs were detected for ethionamide (n = 2; 1.3%) and for the FQ (n = 2; 1.3%). One MDR isolate was identified (Fig. 1).

The detected mutations underpinning these genotypic-based predictions are listed in Table 1 with the Ser315Thr at the *kat*G gene being the most frequent mutation associated with isoniazid resistance (n = 4/6; 66.7%). Two additional *inhA* promoter mutations (*inhA* C-15T and T-8C) were putatively associated with isoniazid resistance and ethionamide resistance. Resistance to rifampicin was driven by two distinct *rpoB* mutations: Ser450Leu (n = 2; 1.3%) and Ser441Leu (n = 1; 0.7%). Two putative compensatory mutations were detected in the single MDR isolate found: *ahpC* − 48G>A and *rpoC* Phe452Ser^19^. The complete resistance-related mutation profile of all isolates is shown in the Supplementary spreadsheet.

**Table 1:** Mutations detected in genes associated with drug resistance across the 151 M*.tb* isolates included in the study.

	Resistant strain	n	Related resistance mutations
Isoniazid	6 (4.0%)	4	*kat*G Ser315Thr
		1	*fab*G1 −15C>T
		1	*fab*G1 −8T>C
		1	*ahp*C −48G>A
Rifampicin	3 (2.0%)	1	*rpo*B Ser441Leu
		2	*rpo*B Ser450Leu
Pyrazinamide	1 (0.7%)	1	*pnc*A Val139Met
Ethambutol	1 (0.7%)	1	*emb*B Met306Val
Ethionamide	2 (1.3%)	1	*fab*G1 −15C>T
		1	*fab*G1 −8T>C
Streptomycin	2 (1.3%)	2	*rps*L Lys43Arg
Fluoroquinolones	2 (1.3%)	1	*gyr*A Asp94Tyr
		1	*gyr*A Ala90Val

### Genomic population structure and phylogeny

All strains had been previously characterized by classical spoligotyping by membrane reverse-hybridization methods, revealing a population structure composed mainly of LAM strains (n = 91; 60.3%), followed by the ill-defined T strains (n = 25; 16.4%) and Haarlem (n = 12; 7.9%). In total, 38 different SITs were found; the SIT 216/LAM5 (13.2%) was the most prevalent, followed by SIT 42/LAM9 (7.3%), SIT 17/LAM2 (7.3%) and SIT 64/LAM6 (7.3%) (Fig. 1). As all clinical isolates were subsequently subjected to WGS, and consistent with the spoligotyping-based classification, all 151 isolates were classified as *M.tb* Lineage 4 (Euro-American) across 13 of its sub-lineages with sub-lineages 4.3.3 (30.5%), 4.3.4.1 (19.9%) and 4.3.4.2 (13.2%) being the most frequent. Moreover, screening for the *fbpC*^103^ SNP marker for LAM confirmed the spoligotyping-based classification of all LAM strains while three isolates initially classified as belonging to the T family were found to bare *fbpC*^103^ and, from a phylogenetic standpoint, should therefore be considered as LAM strains (SC61, SC115, SC134 [SIT823/T1]). Eleven additional isolates without clade assignment or of unknown profile on SITVIT2 also carry this marker (ten Orphans and one SIT2752) (Fig. 1). A genome-wide phylogeny based on 17,027 core SNPs displayed a topology congruent with the distribution of the different sub-lineages found where the LAM strains herein assessed based on the *fbpC*^103^ polymorphism formed a deeply rooted monophyletic clade. All drug resistant isolates detected were scattered across the phylogenetic tree suggesting independent emergence of drug resistance associated mutations.

### SNP-based clustering and M.tb transmission

**Figure 2:**
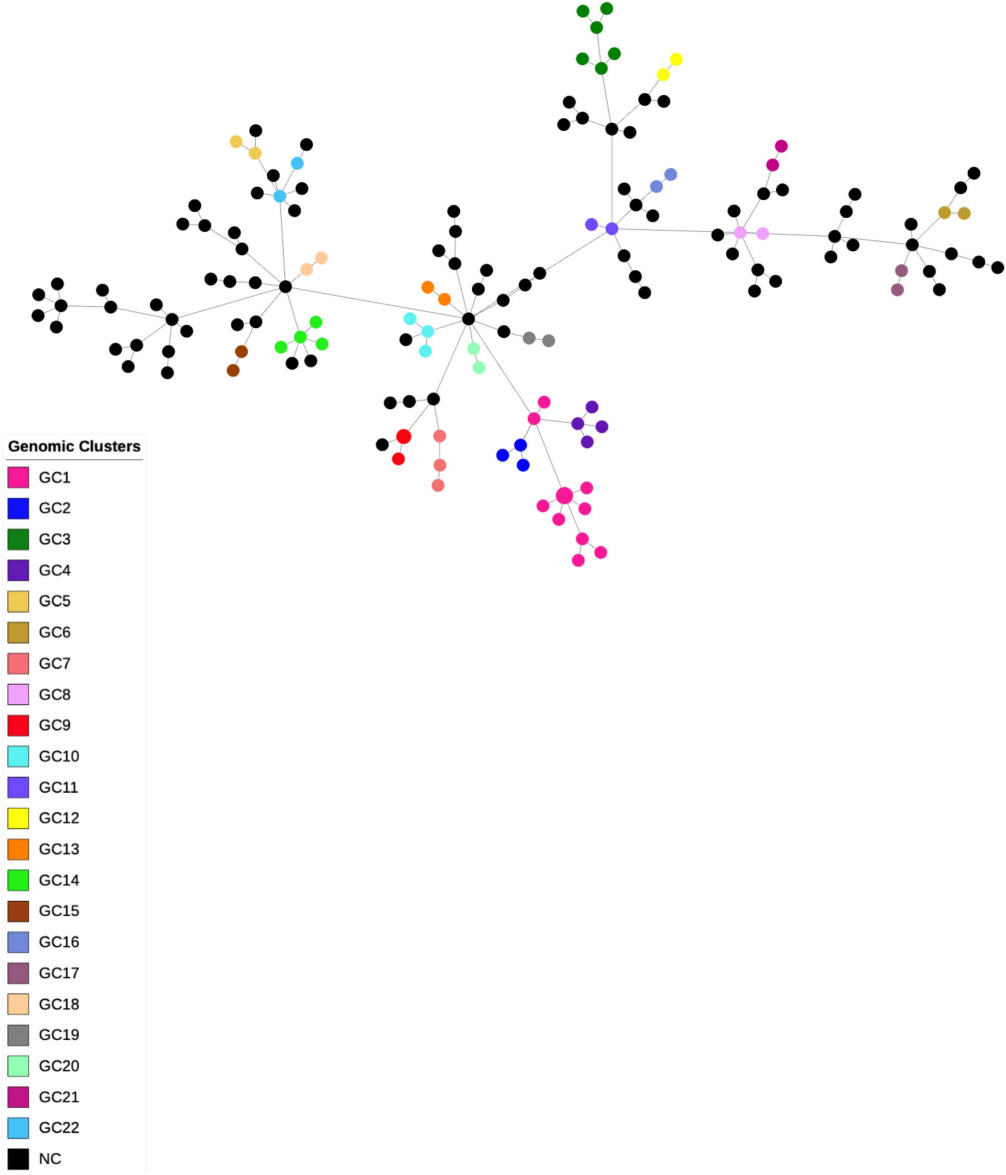
Minimum spanning tree (MST) of the 151 *M.tb* clinical isolates included in the present study. This MST is based on 17,027 core SNPs and nodes are shown coloured in function of the associated genomic cluster.

The average distance amid the core-genome of the 151 isolates was 420.3 SNPs. To delineate genomic clusters that might reflect recent transmission underpinned by putative epidemiologically linked patients, isolates within a maximum distance of 5 SNPs were assigned to genomic clusters (GC). Using this cutoff criterion, a total of 66 (43.7%) isolates were grouped into 22 clusters. The largest cluster (GC1) involved 12 isolates including one isolate obtained from a patient that spent time in a prison. The second largest cluster (GC3) involved six patients while the third largest clusters (GC14 and GC4) included four isolates each; GC14 with one isolate from an individual with incarceration history and GC4 including two epidemiologically-linked patients that shared the same household (pairwise distance: 0 SNPs). The remaining 18 genomic clusters included 42 isolates: one cluster included one isolate from an individual that spent time in a prison establishment; another cluster included a homeless patient; and, one other cluster was composed of two household contacts (pairwise distance: 0 SNPs; Supplementary Table S2). All clusters except GC1, GC8 and GC22 formed monophyletic branches in the phylogenetic tree suggesting further circulation and differentiation of strains within the same branch. The GC2 branch stemmed from within the GC1 clade. GC5 (n = 2) and GC22 (n = 2) isolate appear to have emerged from a broader late-branching phylogenetic SIT73/T (sub-lineage 4.1.2) clade that may represent a group of a mostly non-clustered strain that underwent extensive circulation in the community. A minimum spanning tree (MST) obtained for all isolates confirmed the integrity of all clusters highlighting a more extensive dissemination of GC1 isolates along with the observation of independently generated drug resistance associated nodes in the tree (Fig. 2, Supplementary Figure S1).

**Figure 3:**
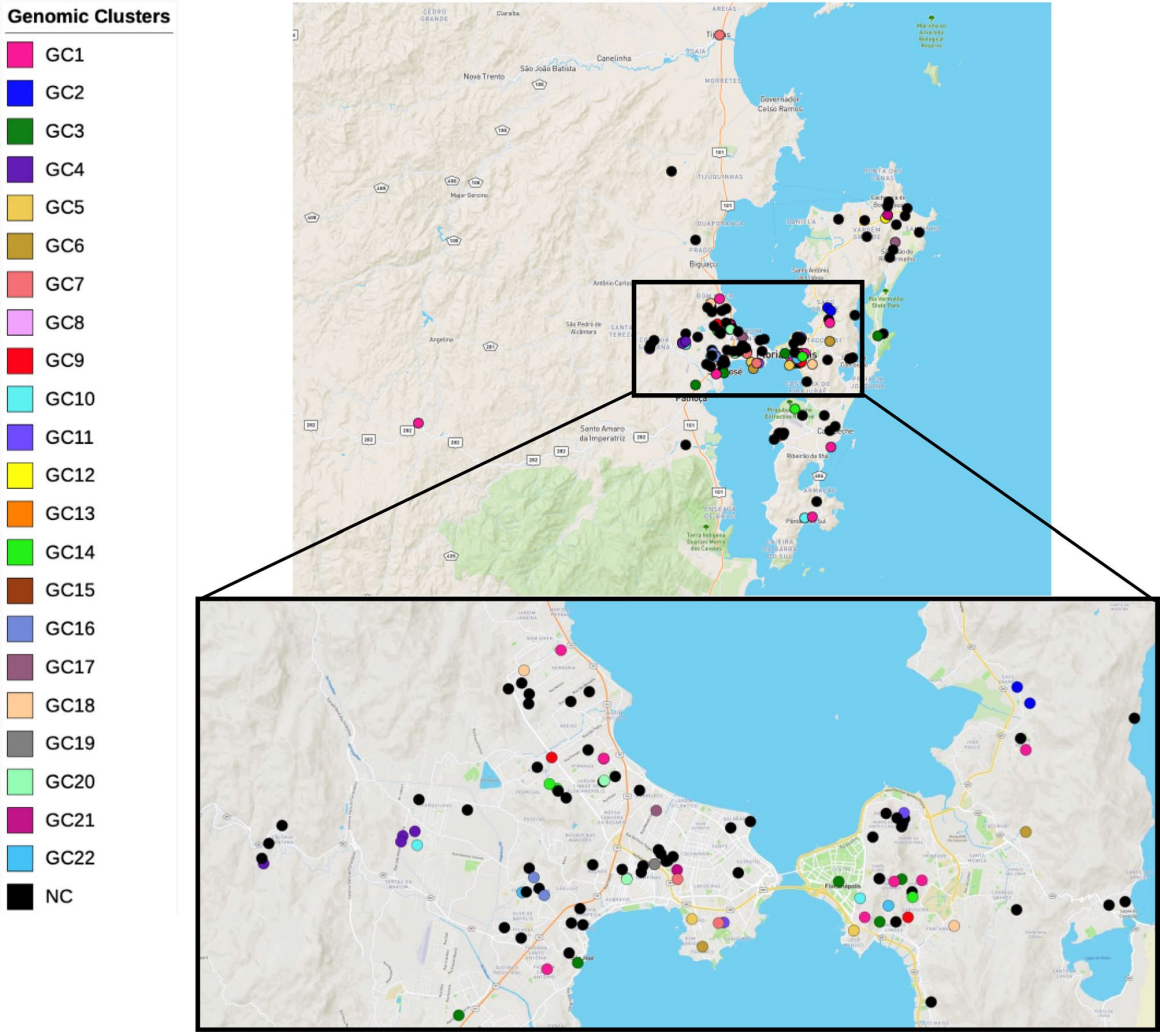
Geographic distribution of 142 out of the 151 TB cases (excluding 9 homeless) included in the study for which address of residence was available. These cases were geographically mapped according to the respective address of residence and are shown in the map colored according to the respective isolate’s genomic cluster. The spatial distribution of these cases and correlation with the genomic cluster shows an extensive geographical spread of the larger clusters (n ≥ 3) except for GC2, GC4 and GC10. The map was created using the online microreact tool, available at https://microreact.org/project/hrdK7GcztX8M2ZXEXjc579.

In the present sampling, the geographical distribution of patients across metropolitan region by residence location showed a higher concentration of cases among patients living at the central region of Florianópolis. Nevertheless, genome clustered cases did not show any particular pattern of geographical clustering as patients whose isolates were clustered showed a wide geographical dispersion in the studied region (Fig. 3). Comparing the pairwise geographic distances between patients’ residence (Fig. 3, Supplementary Figure S2) no statistically significant difference was observed between non-clustered patients (average pairwise distance: 13.7 km; range 0–45 km) and patients within the seven largest clusters (n ≥ 3 patients) except for: patients in GC2 (average pairwise distance: 3.7 km; range 1–5 km) which showed a statistically lower pairwise distance (*p* = 0.0302, Wilcoxon Rank Sum Test); patients in GC4 (average pairwise distance: 2.2 km; range 0–5 km) which also showed a statistically lower average pairwise geographic distance (*p* = 0.0004, Wilcoxon Rank Sum Test); and, likewise for patients in GC10 (average pairwise distance: 2.2 km; range 0–5 km; *p* = 0.0004, Wilcoxon Rank Sum Test). The geographical distribution of the analysed cases is similar to the 263 cases reported for Greater Florianópolis in the same period (Supplementary Figure S3).

### Treatment outcome and statistical association

According to the data collected, 107 (70.9%) patients were declared cured, treatment failure occurred in four (2.6%) patients and six (4.0%) patients died. Additionally, treatment dropout was observed for 33 (21.9%) patients and no information on the treatment outcome was available for one (0.7%) patient (Table1).

We found a statistically significant association between TB outcome and HIV coinfection (*p* = 0.022, Fisher's exact Test) since the coinfected patients had a statistically lower cure rate (75% vs 94.5%, respectively) and a higher death rate (18.7% vs 2.2%, respectively). No statistical association between TB outcome and the others risk factors (alcohol intake, illicit drugs usage and clustering) was found (*p* ≥ 0.05; Table 2).

**Table 2:** Risk factors associated with TB outcome, treatment dropout and hemoptysis.

Risk factor	TB outcome					Treatment dropout				Hemoptysis			
	Death	Cure	Treatment failure	Total	*p*	No	Yes	Total	*p*	No	Yes	Total	*p*
**Alcohol intake**													
No	3 (4.9%)	55 (90.2%)	3 (4.9%)	61		61 (83.6%)	12 (16.4%)	73		44 (63.8%)	25 (36.2%)	69	
Moderate	0 (0.0%)	23 (100.0%)	0 (0.0%)	23	0.230	23 (85.2%)	4 (14.8%)	27	**0.009**	17 (63.0%)	10 (37.0%)	27	0.813
Excessive	3 (13.6%)	18 (81.8%)	1 (4.6%)	22		22 (59.5%)	15 (40.5%)	37		23 (69.7%)	10 (30.3%)	33	
**HIV coinfection**													
Pos	3 (18.7%)	12 (75%)	1 (6.3%)	16	**0.022**	16 (59.3%)	11 (40.7%)	27	**0.009**	21 (87.5%)	3 (12.5%)	24	**0.011**
Neg	2 (2.2%)	85 (94.5%)	3 (3.3%)	90		90 (82.6%)	19 (17.4%)	109		63 (60.0%)	42 (40.0%)	105	
**Illicit drug usage**													
Yes	2 (8.3%)	22 (91.7%)	0 (0.0%)	24	0.464	24 (51.1%)	23 (48.9%)	47	**0.000**	32 (71.1%)	13 (28.9%)	45	0.290
No	3 (3.7%)	73 (91.3%)	4 (5.0%)	80		80 (90.9%)	8 (9.1%)	88		50 (61.7%)	31 (38.3%)	81	
**Clustering**													
Yes	2 (3.9%)	46 (90.2%)	3 (5.9%)	51	0.391	51 (78.5%)	14 (21.5%)	65	0.905	42 (66.7%)	21 (33.3%)	63	0.830
No	4 (6.1%)	61 (92.4%)	1 (1.5%)	66		66 (77.6%)	19 (22.4%)	85		50 (64.9%)	27 (35.1%)	77	

Statistically significant associations are highlighted in bold.

Regarding treatment dropout and risk factors, we found that: higher treatment dropout rates were statistically associated with excessive alcohol intake (*p* = 0.009, Chi-square Test) and HIV coinfection (*p* = 0.009, Chi-square Test); HIV coinfection was associated with illicit drug usage (p ≤ 0.001, Chi-square Test), alcohol intake (p ≤ 0.001, Chi-square test) and lower rates of hemoptysis (*p* = 0.011, Chi-square Test; Table 2). Illicit drug usage and excessive alcohol intake were also found to be associated (p ≤ 0.001, Chi-square Test). No statistically significant association was found between clinical characteristics and SNP-based *M.tb* lineages. We tested also the hypothesis of a possible association between the outcomes and/or clinical characteristics and clustering, however, no association was found. Likewise, no significant association was found between risk factors and clustering (data not showed).

## Discussion

This study evaluates the scenario of *M.tb* strain diversity between May-2014 and May-2016, analyzing individuals from the metropolitan region of Florianópolis diagnosed with pulmonary TB at Florianópolis and São José (the two most populous of the 22 cities that comprise the metropolitan region of Florianópolis). Despite the importance of knowing the molecular profile of circulating *M.tb* strains in a given setting, the existing data for this particular region is limited to the classical genotypic characterization of circulating strains^17,18,20^. The present study comprises the first genome-wide study on *M.tb* in Santa Catarina state. Greater Florianópolis has a higher TB incidence when compared to the overall incidence rate on the state level, an intermediate prevalence setting^21^. In this study sample we found a high rate of TB-HIV co-infection (19.71%) that is consistent with the rate expected for this region (22.6%) and is well above the national rate (9.4%)^22^. Co-infected patients were associated with a low cure rate and excessive consumption of alcohol and drugs. HIV-coinfection is a widely known risk factor for TB development since the risk for TB development is 19 times higher in the population living with HIV when compared with the rest of the population, leading to poorer outcomes and lower relapse-free cure rates^1,23^. Nonetheless, alcohol abuse or unhealthy alcohol usage is an increasingly acknowledged risk factor known to affect the outcome of TB treatment and a risk factor for TB treatment adherence^24,25^. Recent data obtained in Uganda and Kenya further demonstrate that alcohol use among HIV-infected individuals appears to be associated with decreased viral suppression due to the lower diagnosis rate and lower likelihood of being on an anti-retroviral treatment regimen if already HIV diagnosed^26^. In this regard, an integrated approach to reduce unhealthy alcohol consumption or illicit drug usage may lead to better outcomes^27^.

WGS data enabled the screening of drug-resistance conferring mutations on a genome-wide scale which allowed the identification of 10 (6.6%) isolates resistant to at least one anti-TB drug and one (0.7%) MDR-TB isolate. Despite the low MDR-TB rate in this study, two clinical isolates from different patients are predicted to be mono-resistant to the FQs due to two distinct high-confidence *gyrA* mutations for FQs resistance prediction^16,28^. FQ mono-resistance or among non-MDR isolates has been reported across multiple settings with varying ranges^29^. Recently Kim et al.^30^ reported a 0.8% FQ resistance rate among non-MDR strains detected across multiple hospitals in South Korea while revealing an increasing trend over the last two decades. TB patients with prior FQ prescription to TB diagnosis, usually to treat community-acquired pneumonia, have a three-fold higher risk of having FQ-resistant TB^31^. Multiple FQ prescriptions, FQ prescription more than 60 days prior for TB diagnosis and for more than 10 days are associated with FQ-resistant TB^31,32^. This study shows a 1.3% FQ-resistance rate among non-MDR-TB patients for this setting driven by two independent mutational events. One limitation to the interpretation of the data lies in the fact that no data regarding previous FQ exposure was obtained for these two patients and, as such, we cannot exclude these from being primary FQ mono-resistant TB cases^33^. Nonetheless, the detection of these two FQ mono-resistant isolates warns against a possible excessive usage of FQs in the community and calls upon additional antimicrobial stewardship measures since to our knowledge these comprise the first two FQ mono-resistance cases to be reported in Brazil.

Regarding *M.tb* diversity, SNP-based typing classified all the 151 isolates as Euro-American lineage 4 strains. The Euro-American lineage predominance in Santa Catarina State and in the Southern Brazil^17,34^, occurred due to migratory processes from Europe to South America that increased in the seventeenth century^35^. Therefore, LAM, T and Haarlem, the most common spoligotyping families identified in this study, were also found in other studies in Southern Brazil^17,20,34,36,37^. Our examination of the distribution of the fbpC^103^ SNP, considered as a highly specific marker for the LAM family not only confirmed the identification of all spoligotyping-based identified LAM, but increased the frequency of this lineage to 69.5% (105/151). The three SIT823/T1 isolates, assigned to the “ill-defined” T family according to SITVIT2 are in fact phylogenetically positioned as LAM in the phylogenetic tree.

In accordance with previous studies^38,39^, using a conservative five-SNP threshold to define genomic transmission clusters enabled the clustering of 43.7% of the isolates included in the study thereby demonstrating that approximately half of the TB cases in metropolitan region between 2014 and 2016 were due to recent transmission. One recent epidemiological transmission study conducted in the Pará State, in North Brazil, across household contacts did find two cases in the same household whose isolates are described as being 9 SNPs apart^40^. In this latter study, the route of transmission is unclear since the temporal distance between these isolates was 7 years and therefore questions if the transmission between these cases was direct and, if missing links are likely to exist. Herein, we opted to use a 5 SNP distance cutoff as to obtain high-confidence genomic clusters that are driven by recent transmission. Also, no association was found between clustering and risk factors, treatment dropout and clinical characteristics when using 12 or 25 SNPs cutoff distances to define clusters (data not shown).

The recent transmission scenario herein obtained lends support to a continued TB transmission, most likely still ongoing. A limitation to the study relies in the fact that only 151 (57.4%) cases were analyzed from a total of 263 reported cases for the same period in Greater Florianópolis and missing links in transmission chains are therefore likely to exist. However, the data conveyed has already enabled the detection of one large transmission cluster (GC1) that is responsible for 7.9% of the cases analyzed. All the isolates grouped into GC1 belonged to *M.tb* sublineage 4.3.3 and to SIT216/LAM5. Interestingly, SIT216/LAM5 has been described in Santa Catarina state as the most prevalent SIT in prison establishments but only one isolate in GC1 had spent time in prison^18^. These facts highlight the epidemiological importance of SIT216/LAM5 clones, now at the community level, and its association with recent transmission. LAM5 spoligotype is also the most reported in Rio Grande do Sul, another state in Southern Brazil, however belonging to SIT93^34^. A parallel situation has been reported with SIT863 MDR-TB strains in Rio Grande do Sul, that was initially identified in prison establishments but is now responsible for the majority of MDR-TB cases in that state^37,41^. The presence of isolates from inmate individuals in the TB transmission chains supports the dissemination of strains between the general population and prison establishments and although the directionality is unclear at this point, the latter along with the entire prison system may act as reservoir of specific strains and promote a wider dispersion of specific clones^42^. Herein, the GC1 cluster showed a widespread geographical distribution when patient residence is considered (Supplementary Figure S2). Additionally, the low core-genome SNP distances among the isolates in these largest genomic clusters, may indicate that transmission has occurred in a very recent timeframe and is likely ongoing, suggesting that in order to prevent further spread of these strains, closer surveillance of these phylogenetically distinct clades is highly important.

Here we conducted the first WGS-based *M.tb* diversity study in SC state. Our study has some limitation, among them, for several samples phenotypic DST were not available, thus, we used WGS data to drug resistance prediction. Besides that, the present study only included new TB cases that started a first-line anti-TB treatment. Although the sampling in the present study occurred from 2014 to 2016, more recent data show an increase in the incidence TB rate (43.5% in 2016–46.0% in 2018)^2,22^ and it would be interesting to evaluate if this is due to strains belonging to the main transmission chains herein identified. This fact reinforces the importance of molecular epidemiology as an instrument for TB surveillance and supporting public health measures.

## Conclusions

The present study stresses the importance of WGS-based approaches that enable high-resolution phylogenetic analysis to investigate *M.tb* transmission in Brazil and when compared with classical genotyping techniques that may lead to overestimated clustering rates^43^. In this study we undertook a WGS-based analysis of the genetic diversity and recent *M.tb* transmission in a Southern Brazil region improving the knowledge about TB dynamics in this setting and filling out the lack of genomic data on *M.tb* strains circulating in the state of Santa Catarina. The data shows that uncontrolled TB transmission in the metropolitan region of Florianópolis occurred and provides precise data to support TB control measures in this region.

## Materials and methods

### Study design and population

A total of 151 M*.tb* complex strains obtained in the Central Laboratory of the State of Santa Catarina (LACEN), the reference laboratory for TB diagnosis in Santa Catarina were included in this study. The samples were from patients with bacteriological confirmation of pulmonary TB, diagnosed in health units at Florianópolis and São José cities (Florianópolis metropolitan region) within a 2-year period (May-2014–May-2016). The study included individuals that started the first-line anti-TB treatment and patients that met the inclusion criteria and agreed to participate were invited to sign the informed consent. Patient enrollment and medical record review were carried out in health units (Hospital Universitário da Universidade Federal de Santa Catarina and primary health units from Florianópolis and a specialized outpatient clinic responsible for TB care in São José city), and an additional epidemiological questionnaire was applied in an interview. Patients with previous anti-TB treatment history were excluded from the study.

The estimated population in the both cities is 700,000 inhabitants^44^ and the TB incidence rate approximately 46 cases per 100,000 population^21^. During the study period, a total of 263 new pulmonary TB cases with positive culture were notified, of these, 218 were able to contact and accepted to participate in the study; 151 had DNA available for sequencing, representing 57.4% of the total (Supplementary Table S3). *Mycobacterium tuberculosis* was isolated from sputum samples obtained from patients before starting anti-TB treatment. The treatment outcomes were assessed upon treatment completion and defined as cure, treatment failure (persistence of smear microscopy and/or culture positives after the treatment), treatment dropout or death.

### Smear microscopy and culture

The smear microscopy and Ziehl–Neelsen method were performed at the Central Laboratory of the State of Santa Catarina (LACEN), the reference laboratory for TB diagnosis in Santa Catarina according to the recommendations of the Recommendations Manual for TB control in Brazil^45^. Isolation *M.tb* complex was performed in Ogawa–Kudoh solid media according to the same Manual^45^. The identification to the *M.tb* complex level was carried by the presence of the cord factor and by MPT64 protein detection-based immunochromatographic test (SD Bioline Kit, Standard Diagnostics, Inc., Korea).

### DNA extraction

The *M.tb* nucleic acids were extracted from mycobacterial cultures grown on Ogawa–Kudoh solid medium using the Cetyltrimethylammonium Bromide (CTAB) method, as described by van Soolingen et al.^46^, at the Laboratory of Molecular Biology, Microbiology and Serology (LBMMS)-UFSC.

### Spoligotyping

Spoligotyping was carried out as described by Kamerbeek et al.^9^ using commercially prepared membrane (Ocimum Biosolutions, Hyderabad, Telangana, India). Hybridizing fragments were detected by chemiluminescence using peroxidase-labelled streptavidin and ECL detection kit (Amersham Biosciences, Amersham, Buckinghamshire, England, UK). The spoligotypes were classified according to the Spoligotyping International Type (SIT) and families, based on SITVIT2 database (https://www.pasteur-guadeloupe.fr:8081/SITVIT2/). The technique was performed by Scheffer during doctoral thesis^47^.

### Whole genome sequencing (WGS)

The *M.tb* genomic DNA of 151 clinical strains was submitted to WGS. Approximately one microgram of DNA was fragmented using a Q800R2 sonicator (QSonica, Newtown, CT, USA) with the following parameters: 3 min sonication with 15 s pulse on, 15 s pulse off and 20% amplitude. The fragmented DNA was selected by size to target 600–700 bp by fragment separation using the Agencourt AMPure XP beads (Beckman Coulter, Code A63882). DNA Library preparation was performed using the NEBNext Ultra II DNA Library Prep Kit for Illumina (New England BioLabs, Code E7645L). The adapters and 8 bp index oligos based on Kozarewa and Turner^48^ were purchased from IDT (Integrated DNA Technologies, San Diego, CA, USA) and used in place of those supplied in the NEB preparation kit in a dual-indexing approach^49^. Paired-end sequencing (2 × 150 bp) was performed on a NextSeq sequencer from Illumina using either a 300 cycle v2 mid output or high output kit (Illumina, Code FC-404-2003 or Code FC-404-2004) using standard Illumina procedure.

### Bioinformatics analysis

Bioinformatic analysis of raw sequence reads was carried out initially using an in-house pipeline for genome-wide variant calling. Raw reads were trimmed to remove adapter sequences and low quality reads using *Trimmomatic (*v0.33) (parameters: LEADING:3 TRAILING:3 SLIDINGWINDOW:4:20 MINLEN:36)^50^ and the read quality control was performed using *FastQC* (v0.11.7) (https://www.bioinformatics.babraham.ac.uk/projects/fastqc/). Trimmed reads were mapped to the *M*.*tb* H37Rv reference genome (GenBank accession number: NC_000962.3) using *BWA-MEM* (v0.7.16). *Samtools* (v1.9)^51^ was used to convert from SAM to BAM format and sorting of mapped sequences. The quality of the resulting BAM file was checked using *Qualimap*^52^ and *Sambamba* (v0.6.8) was used to mark read duplicates^53^. Variants (SNPs and small INDELs) were called using *Samtools*. Variants were filtered based on the following criteria: mapping quality ≥ 50, base alignment quality ≥ 23 and ≤ 2,000 reads covering each site. Variant functional annotation was performed with *SnpEff* (v4.3)^54^. WGS-based resistance prediction was performed using the command-line version of *TB-Profiler*^55^ (v2.4), using the previously produced BAM files as input complementarily, VCF files were also screened for drug resistance associated variants and, if required, visually inspected in the BAM files. The definition of *M.tb* lineages based in SNP-typing method was performed according to the 62 SNPs barcode proposed by Coll et al. 2014^56^ and implemented in *TB-Profiler*. The *fbp*C^103^ polymorphism (G->A at codon 103) was used to differentiate LAM strains from non-LAM strains^57^.

Phylogenetic analysis was performed using Snippy pipeline v4.3.6 (https://github.com/tseemann/snippy) for variant calling and alignment of all core SNP variants. SNP positions within PE/PPE genes or other repetitive regions associated with low mappability scores were removed from the final core-genome alignment, which was composed of 17,027 positions. A maximum-likelihood phylogenetic tree was generated using the PhyML, applying the generalized time reversible (GTR) model and branch support assessed by the approximate Likelihood Ratio Test (aLRT) as implemented in Seaview^58^. The resulting tree was rooted using *M. canettii* (Genbank accession number: NC_019950.1). A minimum spanning tree was generated using Phyloviz (v2.0) and the therein implemented goeBURST algorithm^59^. We used a 5, 12 and 25 SNPs cut-off to delineate genomic clusters^60^ among the core SNP alignment using R along with the *ape* package and the *hclust* function. The patient address was plotted in a map using the online tool Microreact (www.microreact.org). Pairwise geographical distances between patients were assessed in R using the *Imap* package.

### Statistical analysis

We tested the possible association between TB outcome (favorable: cure or treatment completion versus unfavorable: treatment failure or death), treatment dropout and hemoptysis with the following risk factors: alcohol intake, HIV coinfection, illicit drugs usage and clustering among the 151 individuals with available *M.tb* DNA. Besides, we tested the association among the risk factors. For this purpose, we applied the statistical tests Chi-square and Fisher's exact Test performed in IBM SPSS Statistics v.26.

### Ethical approval

This study was approved by the Human Health Research Ethics Committee of Federal University of Santa Catarina (UFSC), protocol number: 2.054.560 CAAE: 66795917.1.0000.0121. Individuals who agreed to participate in the study signed the Informed Consent Term. The study was performed in accordance with relevant guidelines and regulations.

### Bio-containment measures

Diagnosis activities including cultures of clinical specimens of the *M. tb* complex and DNA extraction were carried out under Biosafety Level 2 (BSL-2) containment with BSL-3 safety equipment and work practices.

## Supplementary information

Supplementary file1.

Supplementary file2.

Supplementary file3.

## Data Availability

*Mycobacterium tuberculosis* genome data were deposited in the NCBI BioProject ID PRJNA599957 (see Supplementary spreadsheet).

## References

[1] WHO (2019). Global Tuberculosis Report 2019.

[2] Brasil. Ministério da Saúde. Ministério da Saúde. Departamento de Vigilância de Doenças Transmissíveis. Boletim Epidemiológico: Brasil Livre da Tuberculose: Evolução dos cenários epidemiológicos e operacionais da doença. (2019).

[3] Couvin D, David A, Zozio T, Rastogi N (2019). Macro-geographical specificities of the prevailing tuberculosis epidemic as seen through SITVIT2, an updated version of the *Mycobacterium tuberculosis* genotyping database. Infect. Genet. Evol..

[4] van Soolingen D (1999). Molecular epidemiology of tuberculosis in The Netherlands: A nationwide study from 1993 through 1997. J. Infect. Dis..

[5] Golub JE (2001). Transmission of *Mycobacterium tuberculosis* through casual contact with an infectious case. Arch. Intern. Med..

[6] Small PM (1994). The epidemiology of tuberculosis in san francisco: A population-based study using conventional and molecular methods. N. Engl. J. Med..

[7] Alland D (1994). Transmission of tuberculosis in New York City. An analysis by DNA fingerprinting and conventional epidemiologic methods. N. Engl. J. Med..

[8] Gagneux S (2013). Current topics in microbiology and immunology. Curr. Top. Microbiol. Immunol..

[9] Kamerbeek J (1997). Simultaneous detection and strain differentiation of *Mycobacterium tuberculosis* for diagnosis and epidemiology. J. Clin. Microbiol..

[10] Van Embden JDA (1993). Strain identification of *Mycobacterium tuberculosis* by DNA fingerprinting: Recommendations for a standardized methodology. J. Clin. Microbiol..

[11] Supply P (2006). Proposal for standardization of optimized mycobacterial interspersed repetitive unit-variable-number tandem repeat typing of *Mycobacterium tuberculosis*. J. Clin. Microbiol..

[12] Kostoff D, Kendall J (2018). A quantitative evaluation ofMIRU-VNTR typing against whole-genome sequencing for identifying *Mycobacterium tuberculosis* transmission: A prospective observational cohort study. Genetica.

[13] Meehan CJ (2019). Whole genome sequencing of *Mycobacterium tuberculosis*: Current standards and open issues. Nat. Rev. Microbiol..

[14] Walker TM (2018). A cluster of multidrug-resistant *Mycobacterium tuberculosis* among patients arriving in Europe from the Horn of Africa: A molecular epidemiological study. Lancet. Infect. Dis..

[15] Cohen KA (2015). Evolution of extensively drug-resistant tuberculosis over four decades: Whole genome sequencing and dating analysis of *Mycobacterium tuberculosis* isolates from KwaZulu-Natal. PLoS Med..

[16] Perdigão J (2020). Using genomics to understand the origin and dispersion of multidrug and extensively drug resistant tuberculosis in Portugal. Sci. Rep..

[17] Nogueira CL (2016). First insight into the molecular epidemiology of *Mycobacterium tuberculosis* in Santa Catarina, southern Brazil. Tuberculosis.

[18] Medeiros TF (2018). Molecular epidemiology of *Mycobacterium tuberculosis* strains from prison populations in Santa Catarina, Southern Brazil. Infect. Genet. Evol..

[19] Perdigão J, Portugal I (2019). Genetics and roadblocks of drug resistant tuberculosis. Infect. Genet. Evol..

[20] Prim RI (2015). xMolecular profiling of drug resistant isolates of *Mycobacterium tuberculosis* in the state of Santa Catarina, southern Brazil. Mem. Inst. Oswaldo Cruz.

[21] Brasil. Ministério da Saúde. TabNet Win32 3.0: TUBERCULOSE—Casos confirmados notificados no Sistema de Informação de Agravos de Notificação-Brasil. https://tabnet.datasus.gov.br/cgi/tabcgi.exe?sinannet/cnv/tubercbr.def (2020).

[22] Brasil. Ministério da Saúde. Ministério da Saúde. Departamento de Vigilância de Doenças Transmissíveis. Boletim Epidemiológico: Indicadores prioritários para o monitoramento do Plano Nacional pelo Fim da Tuberculose como Problema de Saúde Pública no Brasil (2017).

[23] Magis-Escurra C (2017). Treatment outcomes of MDR-TB and HIV co-infection in Europe. Eur. Respir. J..

[24] Fleming MF (2006). Alcohol and drug use disorders, HIV status and drug resistance in a sample of Russian TB patients. Int. J. Tuberc. Lung Dis..

[25] Chaves Torres NM, Quijano Rodríguez JJ, Porras Andrade PS, Arriaga MB, Netto EM (2019). Factors predictive of the success of tuberculosis treatment: A systematic review with meta-analysis. PLoS One.

[26] Puryear SB (2020). Associations between alcohol use and HIV care cascade outcomes among adults undergoing population-based HIV testing in East Africa. AIDS.

[27] Sandgren, A., Lonnroth, K., Piret, V., Pierpaolo, de C. & Aljona, K. Collaborative action on tuberculosis and alcohol abuse in Estonia First report of a demonstration project. WHO Reg. Off. Eur. (2013).

[28] Miotto P (2017). A standardised method for interpreting the association between mutations and phenotypic drug resistance in *Mycobacterium tuberculosis*. Eur. Respir. J..

[29] Zignol M (2016). Population-based resistance of *Mycobacterium tuberculosis* isolates to pyrazinamide and fluoroquinolones: Results from a multicountry surveillance project. Lancet Infect. Dis..

[30] Kim H (2019). Trend of multidrug and fluoroquinolone resistance in *Mycobacterium tuberculosis* isolates from 2010 to 2014 in Korea: A multicenter study. Korean J. Intern. Med..

[31] Devasia RA (2009). Fluoroquinolone resistance in *Mycobacterium tuberculosis* the effect of duration and timing of fluoroquinolone exposure. Am. J. Respir. Crit. Care Med..

[32] Long R (2009). Empirical treatment of community-acquired pneumonia and the development of fluoroquinolone-resistant tuberculosis. Clin. Infect. Dis..

[33] Wang Z (2018). Molecular characteristics of ofloxacin mono-resistant *Mycobacterium tuberculosis* isolates from new and previously treated tuberculosis patients. J. Clin. Lab. Anal..

[34] Salvato RS (2019). First insights into circulating XDR and pre-XDR *Mycobacterium tuberculosis* in Southern Brazil. Infect. Genet. Evol..

[35] Brynildsrud OB (2018). Global expansion of *Mycobacterium tuberculosis* lineage 4 shaped by colonial migration and local adaptation. Sci. Adv..

[36] Salvato RS (2019). Molecular characterisation of multidrug-resistant *Mycobacterium tuberculosis* isolates from a high-burden tuberculosis state in Brazil. Epidemiol. Infect..

[37] Perdigão J (2018). Clonal expansion across the seas as seen through CPLP-TB database: A joint effort in cataloguing *Mycobacterium tuberculosis* genetic diversity in Portuguese-speaking countries. Infect. Genet. Evol..

[38] Walker TM (2014). Assessment of *Mycobacterium tuberculosis* transmission in Oxfordshire, UK, 2007–12, with whole pathogen genome sequences: An observational study. Lancet Respir. Med..

[39] Bjorn-Mortensen K (2016). Tracing *Mycobacterium tuberculosis* transmission by whole genome sequencing in a high incidence setting: A retrospective population-based study in East Greenland. Sci. Rep..

[40] Conceição EC (2018). Analysis of potential household transmission events of tuberculosis in the city of Belem, Brazil. Tuberculosis.

[41] DallaCosta ER (2015). Multidrug-resistant *Mycobacterium tuberculosis* of the Latin American Mediterranean Lineage, Wrongly Identified as *Mycobacterium pinnipedii* (Spoligotype International Type 863 [SIT863]), causing active tuberculosis in South Brazil. J. Clin. Microbiol..

[42] Sacchi FPC (2015). Prisons as reservoir for community transmission of tuberculosis, Brazil. Emerg. Infect. Dis..

[43] Stucki D (2016). Standard genotyping overestimates transmission of *Mycobacterium tuberculosis* among immigrants in a low-incidence country. J. Clin. Microbiol..

[44] IBGE. IBGE cidades. https://cidades.ibge.gov.br/brasil/panorama (2020).

[45] Brasil. Ministério da Saúde. Ministério da Saúde. Departamento de Vigilância de Doenças Transmissíveis. Manual de Recomendações para o Controle da Tuberculose no Brasil. (2019).

[46] van Soolingen D, Hermans PW, de Haas PE, Soll DR, van Embden JD (1991). Occurrence and stability of insertion sequences in *Mycobacterium tuberculosis* complex strains: Evaluation of an insertion sequence-dependent DNA polymorphism as a tool in the epidemiology of tuberculosis. J. Clin. Microbiol..

[47] Scheffer, M. C. Influência de fatores de risco do paciente e de características de *Mycobacterium tuberculosis* no desfecho dos casos novos de tuberculose pulmonar tratados com esquema básico: Uma coorte prospectiva de dois anos na Grande Florianópolis/SC. PhD Thesis. UFSC (2017).

[48] Kozarewa I, Turner DJ (2011). High-throughput next generation sequencing. Genome Biol..

[49] Stone NE (2016). More than 50% of clostridium difficile isolates from pet dogs in Flagstaff, USA, carry toxigenic genotypes. PLoS One.

[50] Bolger AM, Lohse M, Usadel B (2014). Genome analysis Trimmomatic: A flexible trimmer for Illumina sequence data. Bioinformatics.

[51] Li H (2009). The sequence alignment/map format and SAMtools. Bioinformatics.

[52] Cruz LM (2012). Qualimap: Evaluating next-generation sequencing alignment data. Bioinformatics.

[53] Tarasov A, Vilella AJ, Cuppen E, Nijman IJ, Prins P (2015). Genome analysis Sambamba: Fast processing of NGS alignment formats. Bioinformatics.

[54] Cingolani P (2012). A program for annotating and predicting the effects of single nucleotide polymorphisms, SnpEff. Fly (Austin).

[55] Phelan JE (2019). Integrating informatics tools and portable sequencing technology for rapid detection of resistance to anti-tuberculous drugs. Genome Med..

[56] Coll F (2014). A robust SNP barcode for typing *Mycobacterium tuberculosis* complex strains. Nat. Commun..

[57] De Almeida IN (2019). Frequency of the *Mycobacterium tuberculosis* RDRio genotype and its association with multidrug-resistant tuberculosis. BMC Infect. Dis..

[58] Gouy M, Guindon S, Gascuel O (2010). Sea view version 4: A multiplatform graphical user interface for sequence alignment and phylogenetic tree building. Mol. Biol. Evol..

[59] Francisco AP, Bugalho M, Ramirez M, Carriço JA (2009). Global optimal eBURST analysis of multilocus typing data using a graphic matroid approach. BMC Bioinform..

[60] Rosłanowski A, Shelah S, Spinas O (2012). Nonproper products. Bull. Lond. Math. Soc..

