# Author Correction: Genomic epidemiology of *Mycobacterium tuberculosis* in Santa Catarina, Southern Brazil

**DOI:** 10.1038/s41598-020-76386-7

**Published:** 2020-11-02

**Authors:** Mirela Verza, Mara Cristina Scheffer, Richard Steiner Salvato, Marcos André Schorner, Fernando Hartmann Barazzetti, Hanalydia de Melo Machado, Taiane Freitas Medeiros, Darcita Buerger Rovaris, Isabel Portugal, Miguel Viveiros, João Perdigão, Afrânio Kritski, Maria Luiza Bazzo

**Affiliations:** 1 Programa Acadêmico de Tuberculose da Faculdade de Medicina e Complexo Hospitalar HUCFF-IDT, Universidade Federal do Rio de Janeiro (UFRJ), Rua Prof. Rodolpho Paulo Rocco, 255, Ilha do Fundão, Rio de Janeiro, RJ 21941-590 Brazil; 2 Laboratório de Biologia Molecular, Microbiologia e Sorologia, Centro de Ciências da Saúde, Universidade Federal de Santa Catarina (UFSC), Florianópolis, Santa Catarina, Brazil; 3 Programa de Pós-graduaçãoem Biologia Celular e Molecular, Universidade Federal do Rio Grande do Sul (UFRGS), Porto Alegre, Rio Grande do Sul, Brazil; 4 Centro de Desenvolvimento Científico e Tecnológico (CDCT), Centro Estadual de Vigilância Em Saúde, Secretaria Estadual da Saúde do Rio Grande do Sul, Av. Ipiranga, 5400, Porto Alegre, RS 90610-000 Brazil; 5 Setor de Bacteriologia da Tuberculose, Laboratório Central do Estado de Santa Catarina (LACEN-SC), Florianópolis, Santa Catarina, Brazil; 6 iMed.Ulisboa-Research Institute for Medicines, Faculdade de Farmácia, Universidade de Lisboa, Lisboa, Portugal; 7 Unidade de Microbiologia Médica, Global Health and Tropical Medicine, Instituto de Higiene e Medicina Tropical, Universidade Nova de Lisboa, Lisboa, Portugal

Correction to: *Scientific Reports* 10.1038/s41598-020-69755-9, published online 30 July 2020

This Article contains an error in the Discussion section.

“In this study sample we found a high rate of TB-HIV co-infection (19.71%) that is consistent with the rate expected for this region (22.6%) and is well above the national rate (9.4%)^22^.”

should read:

“In this study sample we found a high rate of TB-HIV co-infection (17.9%) that is consistent with the rate expected for this region (22.6%) and is well above the national rate (9.4%)^22^.”

