# Supplementary material for: Genomic epidemiology of Mycobacterium tuberculosis in Santa Catarina, Southern Brazil
Source: Sci Rep. 2020 Jul 30;10:12891. doi: 10.1038/s41598-020-69755-9 (PMC7393130; doi:10.1038/s41598-020-69755-9)

**Genomic epidemiology of Mycobacterium tuberculosis in Santa Catarina, Southern Brazil**

**Mirela Verza, Mara Cristina Scheffer, Richard Steiner Salvato, Marcos André Schorner, Fernando Hartmann Barazzetti, Hanalydia Machado, Taiane Freitas Medeiros, Darcita Buerger Rovaris, Isabel Portugal, Miguel Viveiros, João Perdigão, Afrânio Kritski, Maria Luiza Bazzo.**

**Supplementary Figure S1.** Minimum spanning tree (MST) of the 151 *M.tb* clinical isolates included in the present study. This MST is based on 17,027 core SNPs and nodes are shown coloured in function of the absence and presence of mutations.


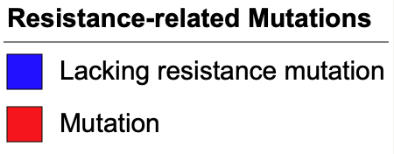


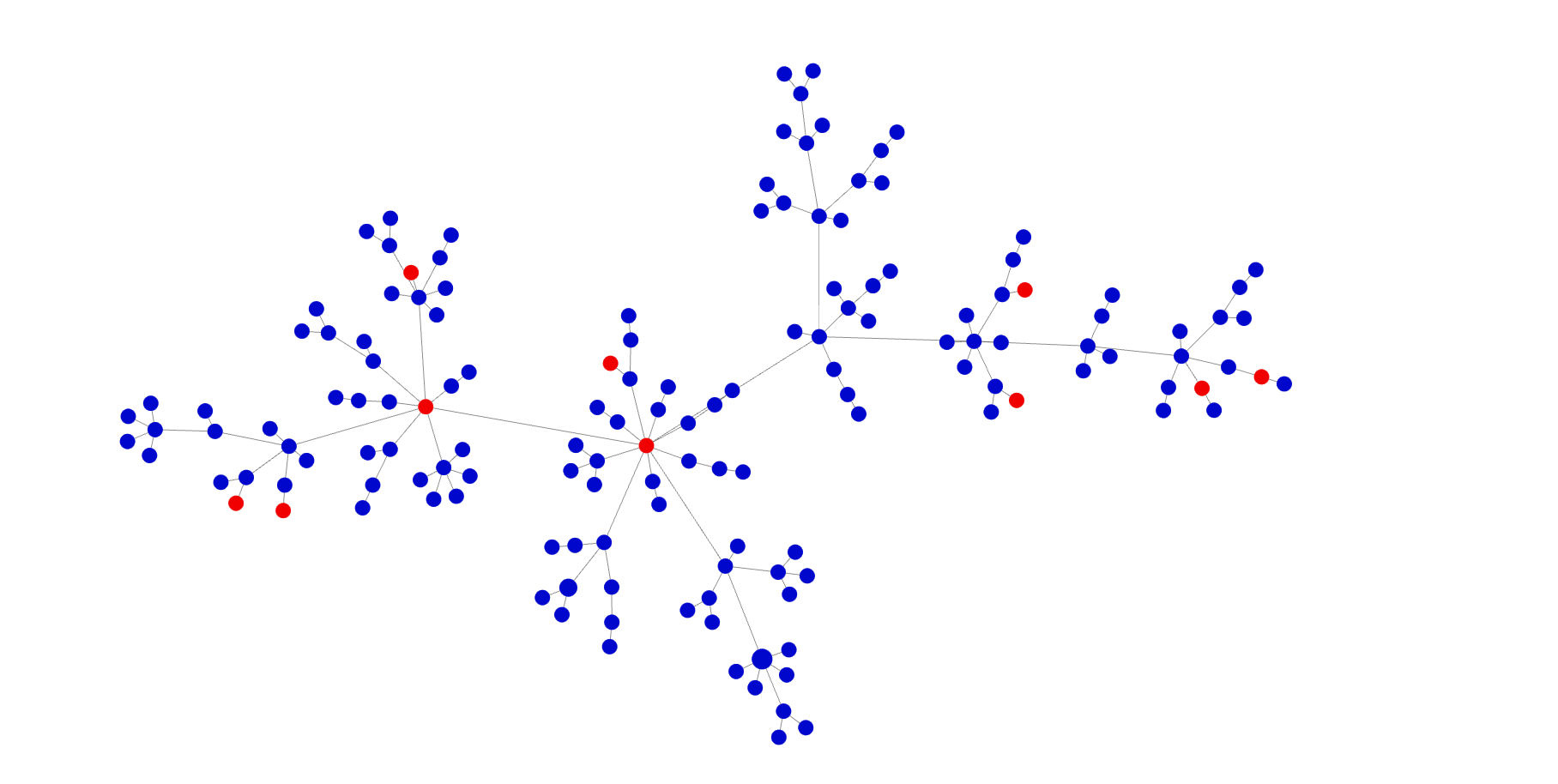


**Supplementary Figure S2.** Pairwise geographical distance between all patients, non-clustered and intra-cluster geographical distances shown either as boxplots (A) or scatter plots (B). The boxplots (panel A) show a non-significant difference between intra-cluster pairwise geographical distances when compared to the non-clustered patient subset. Statistically significant differences at the 0.05 level are highlighted with an asterisk.

**A**


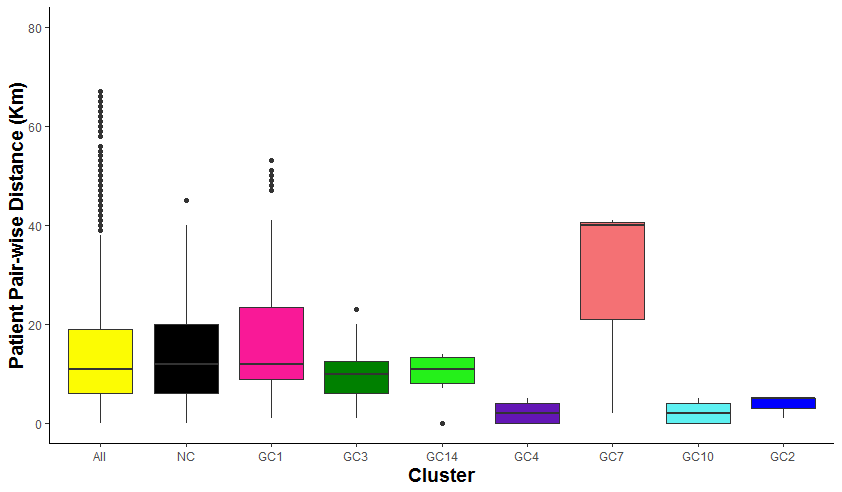


*

*

*

**B**


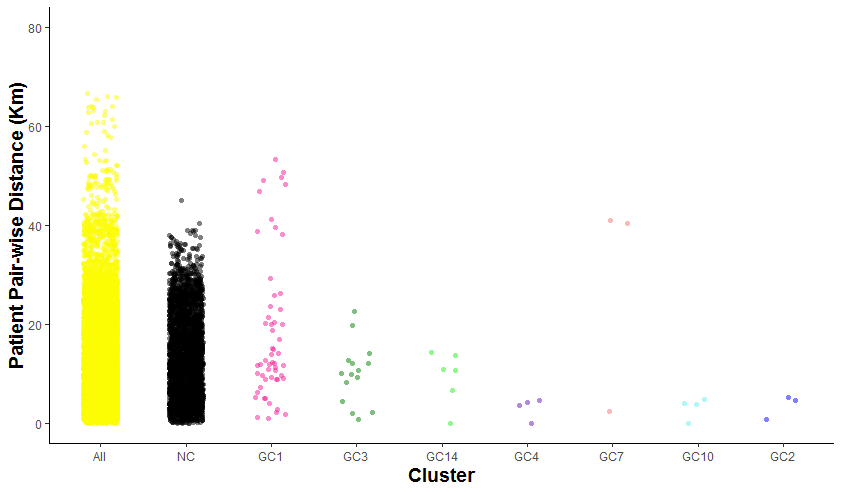


*

*

*

**Supplementary Figure S3. (A)** Geographic distribution of 202 of the 218 individuals (excluding 16 homeless) included in the study and individuals with available DNA. These cases were geographically mapped according to the respective address of residence (*n*=202 in yellow color; *n*=151 in blue color) **(B)** Central area zoom. The map was created using the online microreact tool, available at https://microreact.org/project/knYPddBv1n1cCphgTMRsq9.

**A**


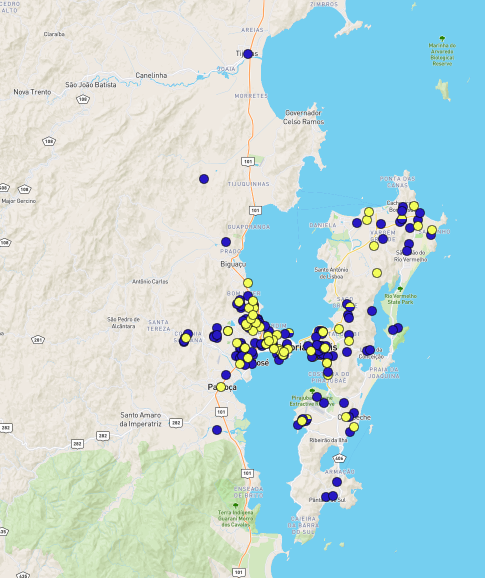


B


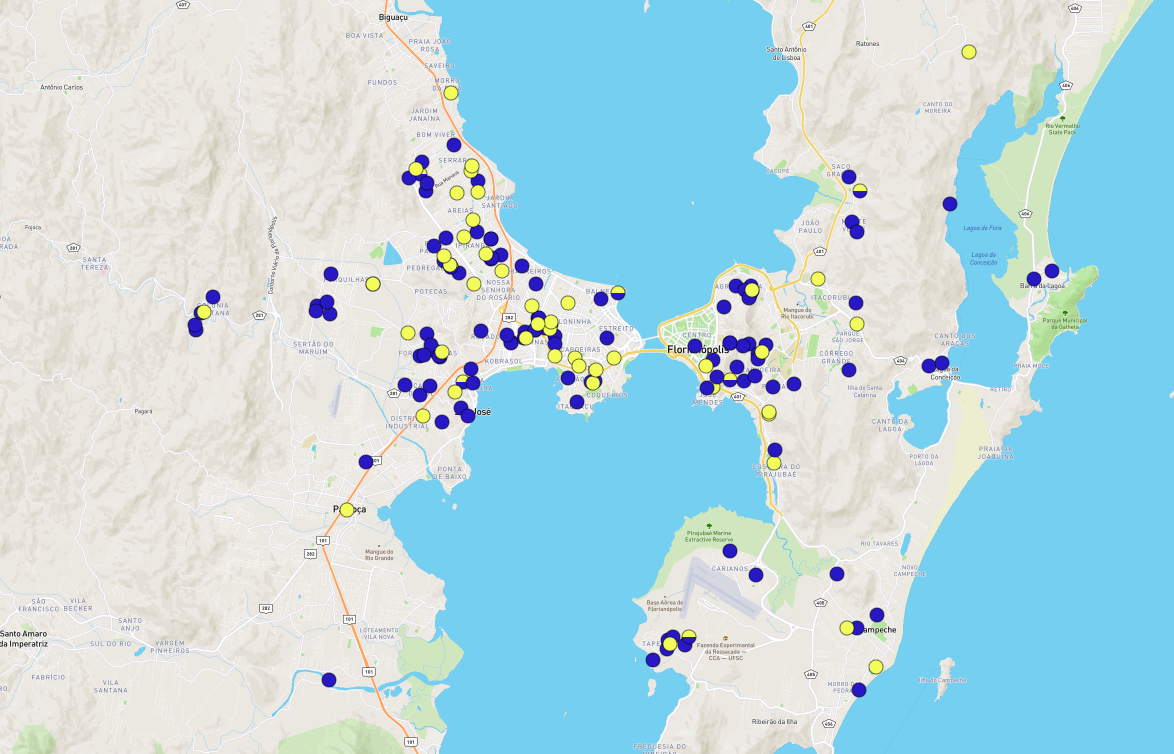

Supplement: Supplementary file 1 — Supplementary file1. [file 41598_2020_69755_MOESM1_ESM.docx]
